# Ventricular Myocardial Deformation in Fetuses With Tetralogy of Fallot: A Necessary Field of Investigation

**DOI:** 10.3389/fcvm.2021.764676

**Published:** 2021-12-10

**Authors:** Xiaoyan Song, Haiyan Cao, Liu Hong, Li Zhang, Meng Li, Jiawei Shi, Juanjuan Liu, Jing Ma, Li Cui, Yi Zhang, Yuman Li, Qing Lv, Mingxing Xie

**Affiliations:** ^1^Department of Ultrasound Medicine, Union Hospital, Tongji Medical College, Huazhong University of Science and Technology, Wuhan, China; ^2^Clinical Research Center for Medical Imaging in Hubei Province, Wuhan, China; ^3^Hubei Province Key Laboratory of Molecular Imaging, Wuhan, China

**Keywords:** fetal echocardiography, tetralogy of Fallot, myocardial deformation, two-dimensional speckle-tracking echocardiography, strain

## Abstract

**Background:** Tetralogy of Fallot (TOF) is one of the most common cyanotic congenital heart defects (CHDs). The patterns of fetal myocardial deformations in TOF have not been well-studied. This study aimed to assess biventricular myocardial deformations in fetuses with TOF compared with normal fetuses.

**Methods:** A retrospective cohort study of fetuses with TOF and gestational age (GA)-matched controls was conducted at a single tertiary referral center from 2014 to 2020. All enrolled fetuses underwent detailed echocardiography, and four-chamber video-clips were recorded and analyzed offline for deformation assessment by using two-dimensional speckle tracking echocardiography (2D-STE). Comparisons for baseline characteristics, cardiac morphological measurements (ventricular, atrial, and great arterial diameters or ratios, global sphericity index), systolic function parameters [ejection fraction (EF), fractional area change (FAC)], and strain parameters [global longitudinal strain (GLS), global longitudinal strain rate in systole and diastole (GLSRs, GLSRd)] were performed between fetuses with TOF and GA-matched controls.

**Results:** Fifty-two fetuses with TOF and 52 GA-matched controls were enrolled in this study. Fetuses with TOF exhibited similar left ventricular (LV) EF (58.51 ± 5.11% vs. 57.59 ± 5.38%, *P* = 0.16) and right ventricular (RV) FAC (43.64 ± 2.89% vs. 44.27 ± 3.04%, *P* = 0.25), compared to normal fetuses. While, in deformational analysis, TOF fetuses demonstrated significantly lower LV and RV GLS values (−22.57 ± 2.91% vs. −27.39 ± 4.38%, *P* < 0.001 for LV GLS; −24.27 ± 3.18% vs. −28.71 ± 4.48%, *P* < 0.001 for RV GLS). Both LV GLS (*r* = −0.518, *P* < 0.001) and RV GLS (*r* = −0.534, *P* < 0.001) were found negatively correlated with the aortic valve-to-pulmonary valve diameter ratio (AV:PV ratio). *Z*-scores of PV annulus and main pulmonary artery (MPA) also had positive correlation with LV and RV GLS, respectively.

**Conclusions:** Decreased biventricular myocardial deformations can appear even in fetuses with TOF with normal systolic ventricular function. Both LV and RV GLS values are correlated with the severity of right ventricular outflow tract obstruction. It indicates 2D-STE may be a more sensitive tool to assess fetal cardiac function than the conventional echocardiographic methods.

## Introduction

Tetralogy of Fallot (TOF), one of the most common cyanotic congenital heart defects (CHDs), accounts for 12–14% of all CHDs and 0.3% of total live births (1). Due to advances in surgical techniques and perioperative management, an increasing number of children with TOF have survived into adulthood in recent decades (2). According to previous studies, the 30- to 40-year survival rates for patients with TOF were 85–90%, and the prognosis was associated with myocardial performance (3, 4).

Fetal TOF is characterized by a series of pathological manifestations, including subaortic ventricular septal defect, aortic-root overriding, and infundibular pulmonary stenosis. However, in contrast to adults, right ventricular hypertrophy is not present in fetuses with TOF (5). With parallel circulation, fetal hemodynamics are distinct from their postnatal occurrences (6). Several studies indicated the fetal ventricle is sensitive to cardiac load changes (7, 8). Obstruction to cardiac outflow in fetuses with TOF might ultimately lead to myocardial impairment *in utero* and may also influence the postnatal outcome and interventional timing.

Studies on myocardial functions in fetal TOF are seldomly seen. Most studies focus on traditional echocardiographic parameters, such as ejection fraction (EF) or fractional area change (FAC). As we know, these measurements may be affected by several factors, such as cardiac size, fetal position, acoustic window, ventricular geometry, etc. However, recent studies have shown that myocardial deformations derived from two-dimensional, speckle-tracking echocardiography (2D-STE) seem to be a more sensitive marker of ventricular dysfunction (9, 10). 2D-STE, a new type of non-invasive, quantitative measurement for evaluating myocardial function, can obtain information directly from 2D video-clips. It is independent of the insonation angle and ventricular geometries; even the irregular right ventricle can be analyzed successfully (11–13). 2D-STE provides new insights into human fetal circulation, especially the evolution of myocardial function in various disease states (14–16).

We hypothesized that fetuses with TOF may present impaired myocardial function, and ventricular myocardial deformations assessed by 2D-STE will be more sensitive than traditional echocardiographic parameters in evaluating the cardiac function of fetuses with TOF. Hence, the purposes of our study were to compare biventricular myocardial deformation of fetuses with TOF vs. normal controls, and to explore the correlations between ventricular strains and conventional echocardiographic measurements.

## Methods

### Study Population

This was a retrospective cohort study performed at our tertiary referral center. Singleton pregnancies with fetal TOF, between 20 and 31 gestational weeks, occurring between January 2014 and December 2020, were enrolled as the TOF group. The prenatal diagnosis of TOF was based on the following observations: a ventricular septal defect, obstruction to right ventricular outflow, and an overriding aorta (an overriding rate <50%). Gestational age (GA)-matched, healthy singletons were selected as the control group. Exclusion criteria for the TOF group included those with additional complex cardiac defects, incomplete clinic information, poor image quality, or loss to follow-up. Fetuses with suspected growth restriction, persistent non-sinus rhythm, maternal disease, or extracardiac anomalies that may alter fetal cardiac function were excluded from both groups. Prenatal diagnosis of TOF was confirmed by postnatal echocardiography or postmortem autopsy. And fetuses in the control group proved to have normal heart according to echocardiography after birth. This study was approved by the Ethics Committee of Tongji Medical College of Huazhong University of Science and Technology.

### Data Collected

Maternal and fetal demographic data were collected. Data including maternal age, body mass index (BMI), pregnancy history, last menstrual period (LMP), gestational age (calculated by ultrasound), fetal weight at screening, additional cardiac defects, extracardiac deformations, chromosomal anomalies, the fetal outcome were recorded.

All the fetuses underwent a second- or third-trimester fetal echocardiography examination. Prenatal echocardiograms were obtained on a GE Voluson E10 (GE Healthcare, Zipf, Austria) ultrasound machine. Doppler and echocardiographic evaluations were performed using a C2-9 (3–9 MHz) or an RM6C (1–7 MHz) transducer. The structural ultrasound of all the cases enrolled included a detailed extracardiac and cardiac examination, following the International Society of Ultrasound in Obstetrics and Gynecology (ISUOG) practice guidelines (17). Conventional 2D and Doppler echocardiographic parameters were obtained.

Conventional cardiac morphometry and hemodynamics included measurements of the global sphericity index (GSI), atrial and ventricular diameters, inner diameters of AV annulus, PV annulus, ascending aorta (AAo), main pulmonary artery (MPA), left and right pulmonary arteries (LPA, RPA), ductus arteriosus (DA) and foramen ovale (FO), peak velocities of PV and AV, and heart rate (HR). Great arteries were measured in systole at their maximum diameters; valve annular diameters were measured at their maximal size (18). Ventricular and great arterial diameters were converted to z-scores based on gestational age by LMP, according to published reports (19).

**Figure 1:**
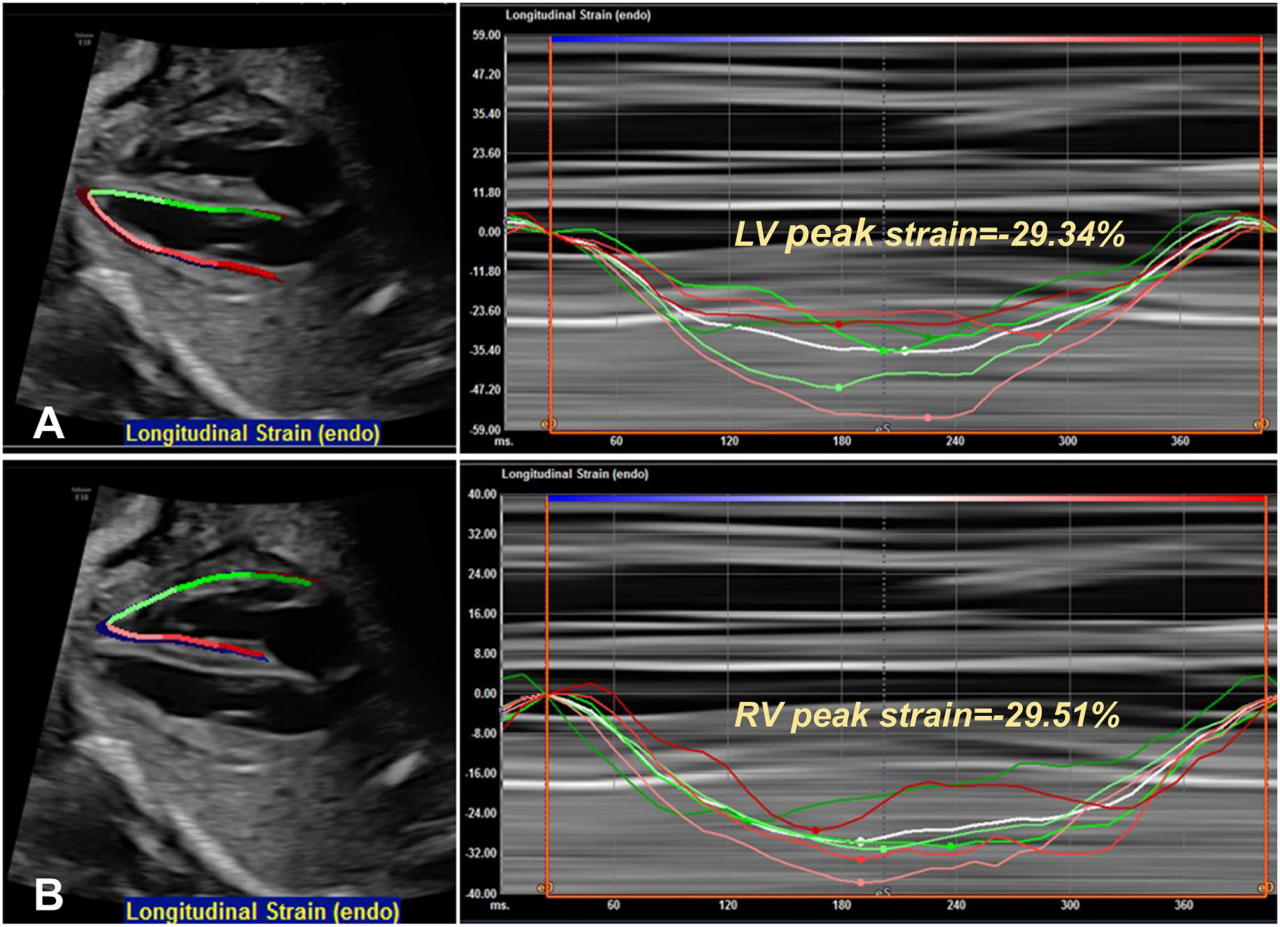
An example of left **(A)** and right **(B)** ventricular deformation by speckle-tracking analysis in one fetus at 26-week gestation. In this case, the peak strain of the left ventricle is −29.34%, and the right ventricle is −29.51%.

Meanwhile, optimized four-chamber cardiac video-clips were acquired for further analysis. The four-chamber view (4CV) images were imported into offline, speckle-tracking analysis software (Cardiac Performance Analysis, TomTec Imaging System, Unterschleissheim, Germany). In the absence of an electrocardiogram, M-mode tracings through the mitral valve were used to define end-systole and end-diastole. Two consecutive end-diastolic frames were selected to analyze a single cardiac cycle. For the ventricular deformation analysis, endomyocardial borders of the ventricle in 4CV were automatically detected with manual adjustment when necessary. Figure 1 depicts examples of left ventricular (LV) and right ventricular (RV) strain curves for fetuses with TOF. The software can calculate the global longitudinal strain (GLS), the global longitudinal strain rate in systole/diastole (GLSRs/GLSRd), EF, and FAC automatically. Strain is reported as a negative number, with lower absolute values indicating worse strain.

Intra-observer variabilities for the determination of LV and RV GLS were assessed in 10 randomly chosen subjects (five for the TOF group and five for the control group) by a single investigator (XY Song), in the same cardiac cycle, at separate times several weeks apart. Inter-observer measurements in the 10 randomly chosen subjects (five for the TOF group and five for the control group) were performed by two reviewers (XY Song and HY Cao) on two separate occasions, each blinded to the other's assessment.

### Statistical Analysis

For statistical data analysis, SPSS 26.0 (IBM) statistical software was used. Descriptive statistics were summarized as frequencies with percentages for categorical values, and medians with interquartile ranges or means ± standard deviation (SD) for continuous variables, depending on the data distribution. Comparison of variables with normal distributions was tested using two-sample *t*-tests. The Wilcoxon rank-sum test was used for non-parametric variables. The correlation between echocardiographic measures and myocardial parameters was tested using a Pearson's correlation coefficient or a Spearman's rank correlation coefficient. Intra- and interobserver reproducibility was tested using intra-class correlation coefficients (ICCs) and Bland-Altman methods. A two-sided *P* < 0.05 was considered statistically significant.

## Results

### Baseline Characteristics

**Figure 2:**
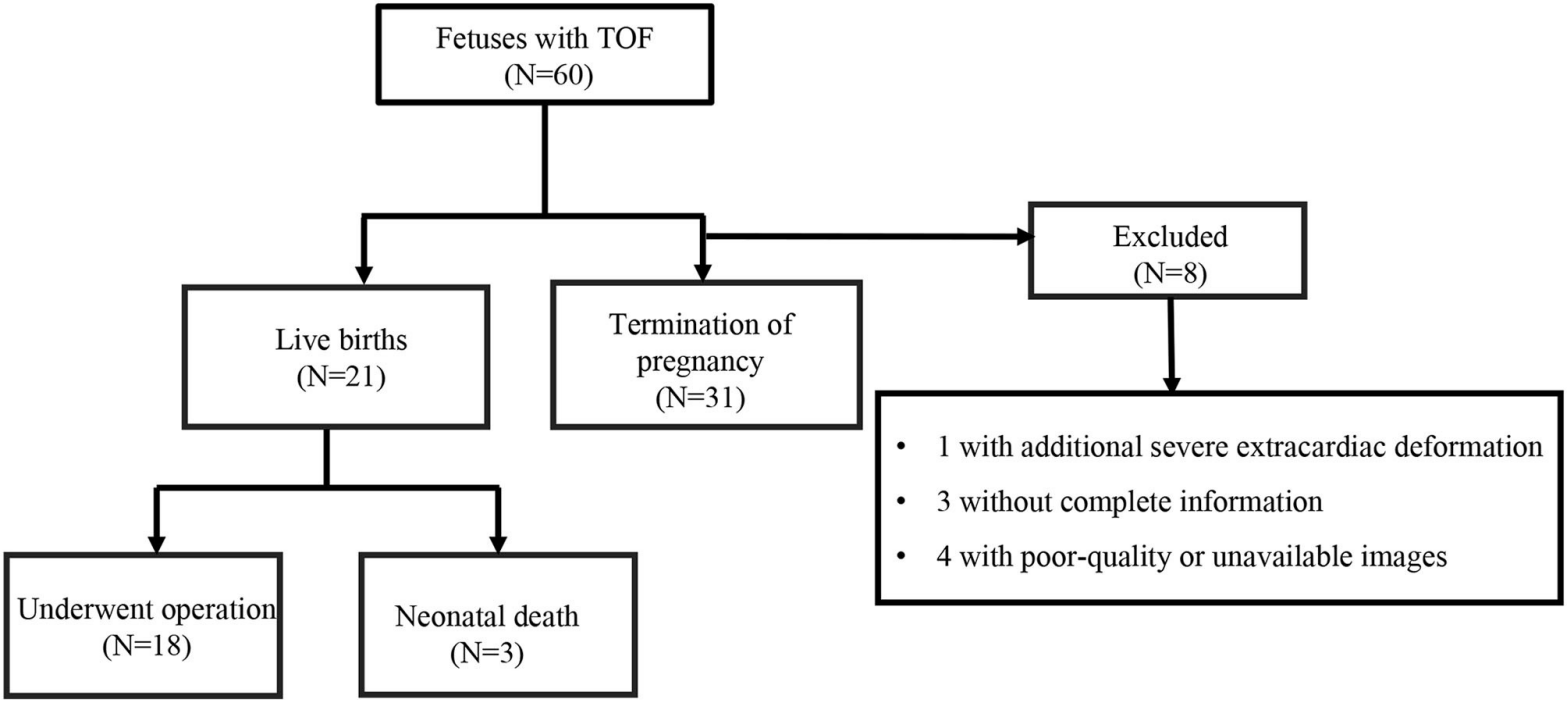
A flow chart showing fetuses with tetralogy of Fallot (TOF) according to the inclusion and exclusion criteria.

The initial study included 60 fetuses with TOF over the specified time frame. Of these, one with an additional, severe extracardiac deformation, three with incomplete information, and four with poor-quality or unavailable images were excluded. Thus, we analyzed 52 fetuses with TOF. Fifty-two GA-matched normal fetuses met the inclusion criteria for this study. Fetal and postnatal outcomes are outlined in Figure 2. There was a termination of pregnancy in 31 (59.6%) fetuses with TOF. Due to the consideration of poor prognosis or poor economic conditions to hardly afford treatment costs, these families made such decisions. Twenty-one (40.4%) fetuses with TOF had a birth with confirmed diagnosis by postnatal echocardiography. There were 18 (18/21, 85.7%) affected infants who underwent operations at the age of 3–12 months, and the remaining three (3/21, 14.3%) live born infants died before the operation could be performed. General maternal and fetal characteristics are summarized in Table 1. No statistically significant differences were observed in maternal ages or BMIs between the TOF and control groups (*P* > 0.05). Estimated fetal weight (EFW) and GA at ultrasound were also similar between the groups (*P* > 0.05). Increased nuchal translucency (NT > 2.5 mm) was present in 3.8% (2/52) fetuses with TOF and only one case (1.9%) in control fetuses. Minor cardiac abnormalities were identified in 40.4% (21/52) of fetuses with TOF: seven with right aortic arch, one with persistent left superior vena cava, five with tortuous arterial duct, and eight with dilated foramen ovale. In the fetuses with TOF, 11.5% (6/52) also had extracardiac anomalies, including three cases with cerebral ventriculomegaly, one case with mild pyelectasis, and two cases with diaphragmatic hernia. Control fetuses demonstrated no intra- or extracardiac anomalies and proved to have structurally normal hearts after birth.

**Table 1:** Baseline characteristics of the fetuses with TOF and normal controls.

**Characteristic**	**Control group**	**TOF group**	***P*-value**
	**(*N* = 52)**	**(*N* = 52)**	
**Maternal characteristics**			
Maternal age (years)	30 (27, 32.8)	29 (27, 32.0)	0.86
BMI (kg/m^2^)	23.9 ± 3.0	23.7 ± 3.4	0.73
Gravidity, *n* (%)			
1	20 (38.5)	12 (23.1)	
2	17 (32.7)	18 (34.6)	
3	5 (9.6)	14 (26.9)	
≥4	10 (19.2)	8 (15.4)	
Parity, *n* (%)			
0	31 (59.6)	25 (4.1)	
1	21 (40.4)	24 (46.2)	
2	0 (0)	3 (5.8)	
**Fetal characteristics**			
GA at US (weeks)	24.4 (23.2, 26.0)	24.4 (23.2, 26.5)	0.84
Estimated fetal weight (g)	644.5 (596.5, 859.3)	653.0 (578.5, 925.8)	0.70
Additional minor cardiac anomalies, *n* (%)			
Right aortic arch	0 (0)	7 (13.5)	
Persistent left superior vena cava	0 (0)	1 (1.9)	
Dilated foramen ovale	0 (0)	8 (15.4)	
Tortuous arterial duct	0 (0)	5 (9.6)	
Extracardiac anomalies, *n* (%)			
Cerebral ventriculomegaly	0 (0)	3 (5.8)	
Mild pyelectasis	0 (0)	1 (1.9)	
Diaphragmatic hernia	0 (0)	2 (3.8)	
Nuchal translucency (>2.5 mm), *n* (%)	1 (1.9)	2 (3.8)	
TOP, *n* (%)	0 (0)	31 (59.6)	

*Data are presented as n (%), mean ± SD, or median (IQR). BMI, body mass index; GA, gestational age; TOP, termination of pregnancy; US, ultrasound*.

### The Myocardial Deformation of LV

**Figure 3:**
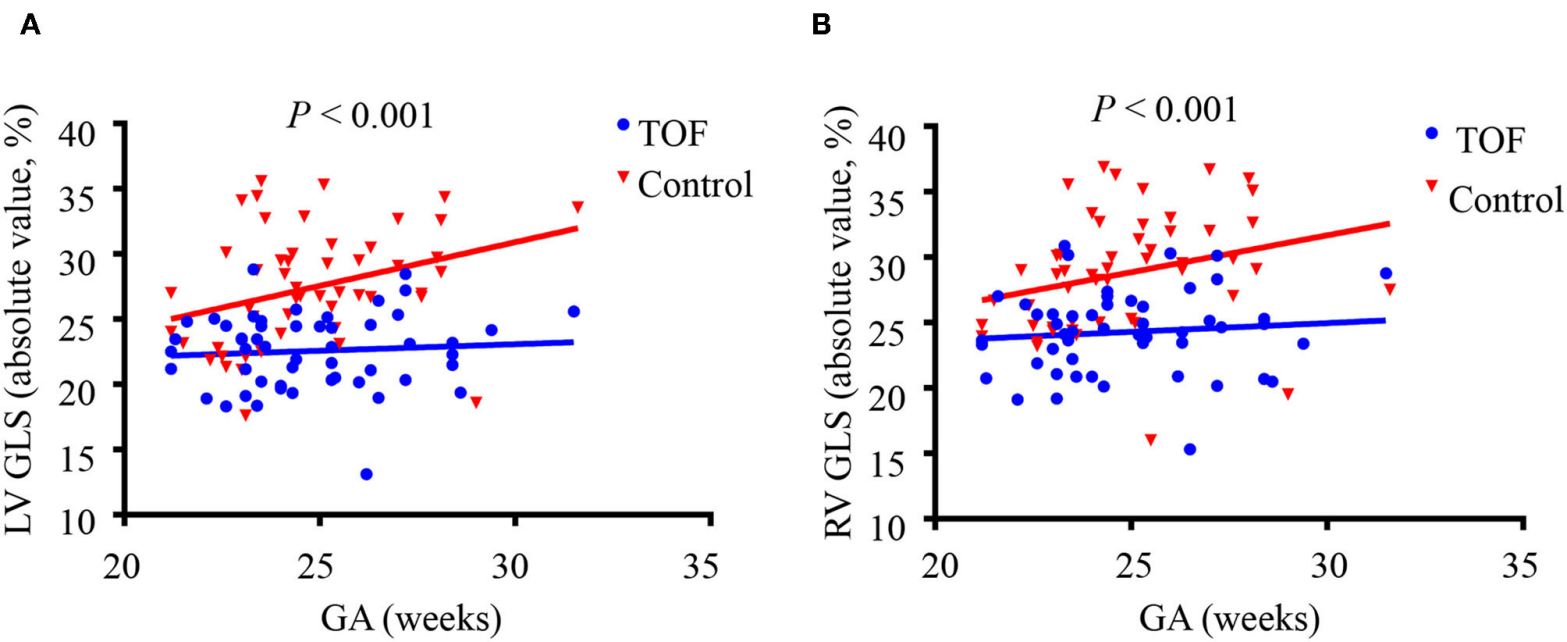
Left ventricular (LV) and right ventricular (RV) global longitudinal strain (GLS) in fetuses with tetralogy of Fallot (TOF) and the control group shown across gestational age. **(A)** LV GLS is decreased in fetuses with TOF across all gestational ages compared to controls. **(B)** RV GLS is decreased in fetuses with TOF across all gestational ages compared to controls. *P*-values represent the difference between fetuses with TOF vs. control fetuses using a two-sample *t*-test.

Compared with control subjects, LV GLS was significantly lower in fetuses with TOF (−22.57 ± 2.91% vs. −27.39 ± 4.38%, *P* < 0.001) (Table 2). This relationship held across all gestational ages as shown in Figure 3A. The strain rates in systole and diastole were also significantly decreased in fetuses with TOF compared to controls (−2.06 ± 0.64 vs. −2.68 ± 0.71 s^−1^, *P* < 0.001 for GLSRs; 1.90 ± 0.93 vs. 2.86 ± 1.22 s^−1^, *P* < 0.001 for GLSRd).

**Table 2:** Myocardial strain by two-dimensional speckle tracking of the left and right ventricles in fetuses with TOF and controls.

**Parameters**	**Control group**	**TOF group**	***P*-value**
	**(*N* = 52)**	**(*N* = 52)**	
**Left ventricle**			
GLS (%)	−27.39 ± 4.38	−22.57 ± 2.91	<0.001
GLSRs (s^−1^)	−2.68 ± 0.71	−2.06 ± 0.64	<0.001
GLSRd (s^−1^)	2.86 ± 1.22	1.90 ± 0.93	<0.001
**Right ventricle**			
GLS (%)	−28.71 ± 4.48	−24.27 ± 3.18	<0.001
GLSRs (s^−1^)	−3.06 ± 0.97	−2.20 ± 0.56	<0.001
GLSRd (s^−1^)	2.94 ± 1.05	2.43 ± 0.83	0.006

*Data are presented as mean ± SD. GLS, global longitudinal strain; GLSRs, global longitudinal strain rate in systole; GLSRd, global longitudinal strain rate in diastole*.

### The Myocardial Deformation of RV

RV GLS was significantly decreased in fetuses with TOF compared to controls (−24.27 ± 3.18% vs. −28.71 ± 4.48%, *P* < 0.001), as shown in Table 2. The difference existed at all gestational ages (Figure 3B). The strain rates of the RV in systole and diastole were also lower in the TOF group (−2.20 ± 0.56 vs. −3.06 ± 0.97 s^−1^, *P* < 0.001 for GLSRs; 2.43 ± 0.83 vs. 2.94 ± 1.05 s^−1^, *P* = 0.006 for GLSRd).

### Conventional Echocardiographic Measurements

Conventional echocardiographic results of fetuses with TOF and controls are displayed in Table 3. Fetuses with TOF revealed similar z-scores of LV and RV end-diastolic diameters (LVEDD, RVEDD), RVEDD: LVEDD ratios, and right atrial (RA): left atrial (LA) transverse diameter ratios, compared to normal controls. However, decreased GSI was observed in the TOF group (1.17 ± 0.04 vs. 1.20 ± 0.05, *P* < 0.001), which indicates more globular-shaped 4CV. Meanwhile, consistent with pathological features, fetuses with TOF manifested enlargement of the diameters of AV annulus and AAo, reduction of the diameters of PV annulus, MPA, LPA, RPA, and DA, and acceleration of pulmonary peak systolic velocity (*P* < 0.001). However, no significant differences were observed between fetuses with TOF and controls regarding traditional ventricular systolic function (58.51 ± 5.11% vs. 57.59 ± 5.38%, *P* = 0.16 for LVEF; 43.64 ± 2.89% vs. 44.27 ± 3.04%, *P* = 0.25 for RVFAC).

**Table 3:** Fetal echocardiographic results in fetuses with TOF and controls.

**Variable**	**Control group**	**TOF group**	***P*-value**
	**(*N* = 52)**	**(*N* = 52)**	
LVEDD *z*-score	0.33 (−0.01, 0.54)	0.13 (−0.45, 0.46)	0.05
RVEDD *z*-score	0.16 (−0.31, 0.52)	−0.05 (−0.51, 0.52)	0.28
RVEDD: LVEDD ratio	1.02 (1.00, 1.06)	1.04 (0.97, 1.09)	0.49
RA: LA transverse diameter ratio	1.13 ± 0.07	1.12 ± 0.12	0.64
AV annulus *z*-score	0.09 (−0.26, 0.52)	1.14 (0.52, 1.76)	<0.001
PV annulus *z*-score	0.04 (−0.36, 0.50)	−2.08 (−3.04, −0.97)	<0.001
AV: PV diameter ratio	0.84 (0.80, 0.88)	1.45 (1.25, 1.64)	<0.001
AAo *z*-score	0.10 (−0.32, 0.37)	1.04 (0.42, 1.72)	<0.001
MPA *z*-score	0.53 (0.11, 0.87)	−2.09 (−3.04, −0.97)	<0.001
LPA *z*-score	0.04 (−0.23, 0.39)	−1.01 (−1.66, −0.28)	<0.001
RPA *z*-score	−0.05 (−0.49, 0.22)	−1.22 (−1.92, −0.69)	<0.001
FO diameter (mm)	0.44 (0.40, 0.49)	0.46 (0.40, 0.56)	0.08
AV PSV (cm/s)	95.4 ± 9.5	94.4 ± 14.9	0.66
PV PSV (cm/s)	70.6 ± 10.9	124.5 ± 36.4	<0.001
LVEF (%)	57.59 ± 5.38	58.51 ± 5.11	0.16
RVFAC (%)	44.27 ± 3.04	43.64 ± 2.89	0.25
GSI	1.20 ± 0.05	1.17 ± 0.04	<0.001
HR (bpm)	147.1 ± 6.0	146.8 ± 7.6	0.84

*Data are presented as mean ± SD or median (IQR). LV, left ventricle; RV, right ventricle; EDD, end-diastolic diameter; LA, left atrium; RA, right atrium; AV, aortic valve; PV, pulmonary valve; AAo, ascending aorta; MPA, main pulmonary artery; LPA, left pulmonary artery; RPA, right pulmonary artery; FO, foramen ovale; PSV, peak velocity in systole; EF, ejection fraction; FAC, fractional area change; GSI, global sphericity index; HR, heart rate*.

**Figure 4:**
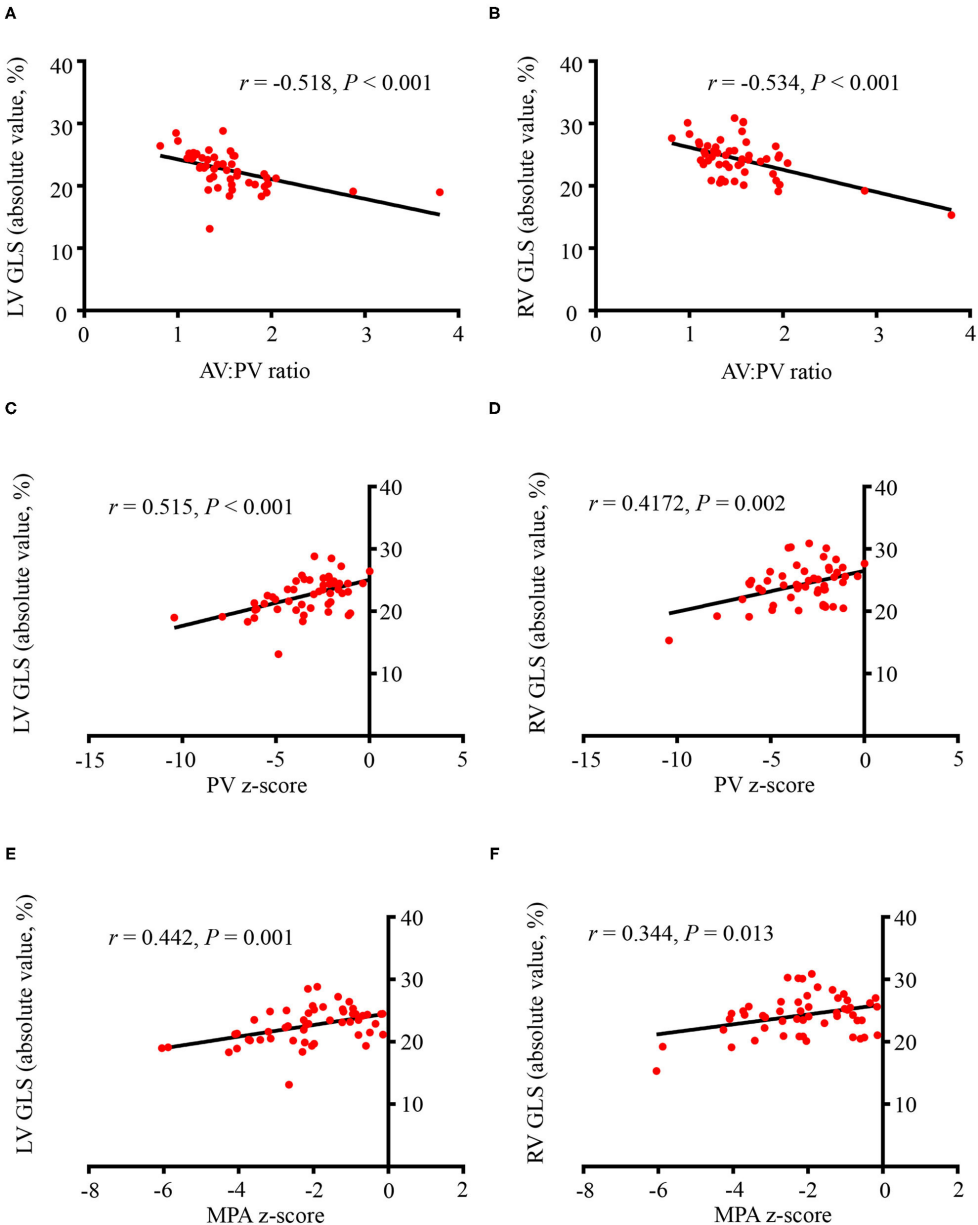
The correlations between conventional parameters and left/right ventricular global longitudinal strains (LV GLS, RV GLS). **(A)** The aortic valve (AV): pulmonary valve (PV) ratio and LV GLS had a modest correlation (*r* = −0.518, *P* < 0.001). **(B)** There was a modest correlation between the AV:PV ratio and RV GLS (*r* = −0.534, *P* < 0.001). **(C)** The PV *z*-score had positive correlation with LV GLS (*r* = 0.515, *P* < 0.001). **(D)** The PV *z*-score had a positive correlation with RV GLS (*r* = 0.417, *P* = 0.002). **(E)** The MPA *z*-score had positive correlation with LV GLS (*r* = 0.442, *P* = 0.001). **(F)** The MPA *z*-score also had positive correlation with RV GLS (*r* = 0.344, *P* = 0.013). All the GLS values were presented as absolute values. GLS, global longitudinal strain; AV, aortic valve; PV, pulmonary valve; MPA, main pulmonary artery.

The correlations between conventional parameters and ventricular deformations in fetuses with TOF were tested (Figure 4). There was a modest correlation between AV:PV ratios and LV GLS (*r* = −0.518, *P* < 0.001) and RV GLS (*r* = −0.534, *P* < 0.001). The PV z-score had positive correlation with LV GLS (*r* = 0.515, *P* < 0.001) and RV GLS (*r* = 0.417, *P* = 0.002). The MPA *z*-score also had positive correlation with LV GLS (*r* = 0.442, *P* = 0.001) and RV GLS (*r* = 0.344, *P* = 0.013). GA, AV z-score, and AAo z-score showed no correlation with LV or RV GLS values (*P* > 0.05).

### Reproducibility of 2D-STE

The measurements obtained by 2D-STE all showed excellent reproducibility. The ICCs and Bland-Altman analyses for the intra- and inter-observer reproducibility of the strain parameters are described in Supplementary Table 1.

## Discussion

This study examined a cohort of fetuses with TOF to explore potentially novel and useful information for counseling families. To our knowledge, this is the first study evaluating ventricular performance metrics in fetal TOF using 2D-STE. This study demonstrates that fetuses with TOF showed preserved LVEF and RVFAC but impaired myocardial strains of both ventricles. The more severe obstruction of the right ventricular outflow tract is, the lower GLS values of both ventricles will be.

Previous studies have demonstrated that Doppler parameters change in obvious ways in fetuses with classic TOF. However, the ventricular function measurements, such as Tei index and fractional shortening (FS), are still in normal ranges (17). Similarly, in our study, it was also observed that LVEF and RVFAC were preserved in fetuses with TOF. However, the reduced global strains observed in this study may suggest impaired myocardial function still exists in fetuses with TOF.

Findings of decreased LV strain in fetal TOF may be due to altered ventricular mechanics in the context of hypertensive RV, abnormal coronary perfusion, and/or intrinsic abnormality of the myocardium. Although many studies have focused on the right ventricular physiology in patients with TOF (20, 21), a study by Wiputra et al. investigated the intracardiac fluid mechanics of fetal TOF in both ventricles (22). They found that LV and RV had similar pressures, but RV presented elevated wall shear stresses (WSS), while LV did not (22). In our study, however, LV also demonstrated altered global myocardial deformations. Our finding is consistent with recent studies on TOF, which indicated that RV cannot be seen in isolation in response to long-term overload (23, 24). In the TOF group, most of the ventricular septal defects (VSDs) are non-restrictive, with a large portion of flow across tricuspid valve shunting to LV. Hence, the pressure of LV and RV is almost equal and affected similarly (22). The LV volume loading will be elevated as a result of an increased right-to-left shunt across the VSD and FO. Therefore, we supposed that subclinical LV myocardial dysfunction has already occurred *in utero*, and manifests as reduced ventricular strain. Our work suggests that the myocardial strain might be a more sensitive indicator than the traditional parameters for assessing fetal cardiac function.

Decreased RV GLS in fetuses with TOF was an expected finding from this study, given the change in RV hemodynamics associated with obstruction of the right ventricular outflow tract, and its consequences on RV development. Such results may be due to adequate adaptation to long-term cardiac load, causing cardiac remodeling, which may change the macro- and microstructure in the developing myocardium (20). In fetal TOF, the chronic pressure overload caused by pulmonary stenosis and increased WSS may modulate myocardial contractility and impact cardiac geometry (21). As presented in this study, decreased GSI in fetal TOF was noted, indicating a more spherical-shaped heart had developed. This shape may allow the heart to maintain a normal stroke volume, and a more globular shape may also reduce the wall stress to help the heart tolerate the pressure overload.

Many prior studies have investigated prenatal echocardiographic markers to predict postnatal surgery timing in fetuses with TOF (25–27). These indicators include reversal of DA flow, PV z-score, and the PV: AV ratio. However, in our study, 18 infants underwent primary repair, while the other three cases had died before intervention. Due to the retrospective nature and the limited sample size of this current study, it is hard to explore the value of conventional or novel indicators in predicting the optimal age of intervention or appropriate surgery approaches. But we hope to conduct a multicentered, prospective cohort study to further explore this in the future.

### Limitations

This is the first quantitative study of ventricular function in fetal TOF using a combination of conventional data and detailed speckle-tracking analysis. However, we recognize there are some limitations to this study. Firstly, this is a single-centered retrospective study. Some cases were excluded due to incomplete information, poor-quality/unavailable images, or extracardiac anomaly, and the selection bias may occur. Secondly, as described above, the high percentage of termination of pregnancy and a limited number of subjects who underwent postnatal surgery make it difficult for us to acquire the true outcome of affected fetuses. Finally, most cases (46/52, 88.5%) in our study were in the second trimester. Hence, the fetuses in early or late pregnancy have not been completely studied. In the future, a large, multicentered, and prospective cohort with a longer postnatal follow-up is needed for further investigation.

## Conclusion

In conclusion, to our knowledge, this report is the largest description of myocardial deformations in fetuses with TOF. Our findings suggest that impaired myocardial ventricular function has already occurred in fetuses with TOF, before conventional cardiac function indicators (like EF and FAC) change. Additionally, strains are correlated with the severity of obstruction to the right ventricular outflow tract. It indicates 2D-STE may be a more sensitive tool to assess fetal cardiac function than the conventional echocardiographic methods.

## Data Availability Statement

The original contributions presented in the study are included in the article/Supplementary Material, further inquiries can be directed to the corresponding author/s.

## Ethics Statement

This study was approved by the Ethics Committee of Tongji Medical College of Huazhong University of Science and Technology. Written informed consent to participate in this study was provided by the participants' legal guardian/next of kin. Written informed consent was obtained from the individual(s), and minor(s)' legal guardian/next of kin, for the publication of any potentially identifiable images or data included in this article.

## Author Contributions

XS, HC, LH, YL, LZ, QL, and MX: conception and design of the study. XS, LC, YZ, JL, and JM: acquisition of data. XS and JS: analysis and interpretation of data. XS and HC: drafting the article. HC: revising the article. QL and MX: finale approval of the article. All authors listed have made a substantial, direct, and intellectual contribution to the work and approved it for publication.

## Funding

This work was supported by grants from National Natural Science Foundation of China (Grant Nos. 81727805, 81922033, and 81771851), the Key Research and Development Program of Hubei (Grant No. 2020DCD015), the Fundamental Research Funds for the Central Universities (Grant No. 5003530082), and the Shenzhen Science and Technology under Grant (Grant No. SGDX20190917094601717).

## Conflict of Interest

The authors declare that the research was conducted in the absence of any commercial or financial relationships that could be construed as a potential conflict of interest.

## Publisher's Note

All claims expressed in this article are solely those of the authors and do not necessarily represent those of their affiliated organizations, or those of the publisher, the editors and the reviewers. Any product that may be evaluated in this article, or claim that may be made by its manufacturer, is not guaranteed or endorsed by the publisher.

## References

[1] Apitz C Webb GD Redington AN. Tetralogy of Fallot. Lancet. (2009) 374:1462–71. 10.1016/S0140-6736(09)60657-7 19683809

[2] Downing TE Kim YY. Tetralogy of Fallot: general principles of management. Cardiol Clin. (2015) 33:531–41. 10.1016/j.ccl.2015.07.002 26471818

[3] Chiu SN Wang JK Chen HC Lin MT Wu ET Chen CA. Long-term survival and unnatural deaths of patients with repaired tetralogy of Fallot in an Asian cohort. Circ Cardiovasc Qual Outcomes. (2012) 5:120–5. 10.1161/CIRCOUTCOMES.111.963603 22235069

[4] Cuypers JAAE Menting ME Konings EEM Opic P Utens EMWJ Helbing WA. Unnatural history of tetralogy of Fallot: prospective follow-up of 40 years after surgical correction. Circulation. (2014) 130:1944–53. 10.1161/CIRCULATIONAHA.114.009454 25341442

[5] Starr JP. Tetralogy of Fallot: yesterday and today. World J Surg. (2010) 34:658–68. 10.1007/s00268-009-0296-8 20091166

[6] Godfrey ME Messing B Cohen SM Valsky DV Yagel S. Functional assessment of the fetal heart: a review. Ultrasound Obstet Gynecol. (2012) 39:131–44. 10.1002/uog.9064 21611999

[7] Kilby MD Szwarc RS Benson LN Morrow RJ. Left ventricular hemodynamic effects of rapid, in utero intravascular transfusion in anemic fetal lambs. J Matern Fetal Med. (2015) 7:51–8. 10.1002/(SICI)1520-6661(199801/02)7:1<51::AID-MFM12>3.0.CO;2-O 9502672

[8] Lewinsky RM Szwarc RS Benson LN Ritchie JWK. Determinants of increased left ventricular output during *in utero* ventilation in fetal sheep. Pediatr Res. (1994) 36:373–9. 10.1203/00006450-199409000-00018 7808835

[9] Gardiner HM Belmar C Tulzer G Barlow A Pasquini L Carvalho JS. Morphologic and functional predictors of eventual circulation in the fetus with pulmonary atresia or critical pulmonary stenosis with intact septum. J Am Coll Cardiol. (2008) 51:1299–308. 10.1016/j.jacc.2007.08.073 18371563

[10] Smiseth OA Torp H Opdahl A Haugaa KH Urheim S. Myocardial strain imaging: how useful is it in clinical decision making? Eur Heart J. (2016) 37:1196–207. 10.1093/eurheartj/ehv529 26508168 PMC4830908

[11] Stricagnoli M Cameli M Incampo E Lunghetti S Mondillo S. Speckle tracking echocardiography in cardiac amyloidosis. Heart Fail Rev. (2019) 24:701–7. 10.1007/s10741-019-09796-z 30989593

[12] Mondillo S Galderisi M Mele D Gameli M Lomoriello VS Zaca V. Speckle-tracking echocardiography: a new technique for assessing myocardial function. JUltrasound Med. (2011) 30:71–83. 10.7863/jum.2011.30.1.71 21193707

[13] Longobardo L Suma V Jain R Carerj S Zito C Zwicke DL. Role of two-dimensional speckle-tracking echocardiography strain in the assessment of right ventricular systolic function and comparison with conventional parameters. J Am Soc Echocardiogr. (2017) 30:937–46.e936. 10.1016/j.echo.2017.06.016 28803684

[14] Chelliah A Dham N Frank LH Donofrio M Krishnan A. Myocardial strain can be measured from first trimester fetal echocardiography using velocity vector imaging. Prenat Diagn. (2016) 36:483–8. 10.1002/pd.4813 26991266

[15] DeVore GR Polanco B Satou G Sklansky M. Two-dimensional speckle tracking of the fetal heart: a practical step-by-step approach for the fetal sonologist. J Ultrasound Med. (2016) 35:1765–81. 10.7863/ultra.15.08060 27353066

[16] Eckersley LG Howley LW van der Velde ME Khoo NS Mah K Brooks P. Quantitative assessment of left ventricular dysfunction in fetal Ebstein's anomaly and tricuspid valve dysplasia. J Am Soc Echocardiogr. (2019) 32:1598–607. 10.1016/j.echo.2019.07.008 31551185

[17] Carvalho JS Allan LD Chaoui R Copel JA Devore GR Hecher K. ISUOG Practice Guidelines (updated): sonographic screening examination of the fetal heart. Ultrasound Obstet Gynecol. (2013) 41:348–59. 10.1002/uog.12403 23460196

[18] Rodenbarger A Thorsson T Stiver C Jantzen D Chevenon M Yu S. Third trimester predictors of interventional timing and accuracy of fetal anticipatory guidance in tetralogy of Fallot: a multi-center study. Prenat Diagn. (2020) 40:870–7. 10.1002/pd.5697 32274817

[19] Schneider C McCrindle BW Carvalho JS Hornberger LK McCarthy KP. Development of Z-scores for fetal cardiac dimensions from echocardiography. Ultrasound Obstet Gynecol. (2005) 26:599–605. 10.1002/uog.2597 16254878

[20] Koestenberger M Nagel B Avian A Ravekes W Sorantin E Cvirn G. Systolic right ventricular function in children and young adults with pulmonary artery hypertension secondary to congenital heart disease and tetralogy of Fallot: tricuspid annular plane systolic excursion (TAPSE) and magnetic resonance imaging data. Congenit Heart Dis. (2012) 7:250–8. 10.1111/j.1747-0803.2012.00655.x 22494699

[21] Egbe AC Taggart NW Reddy YNV Sufian M Banala K Vojjini R. Assessment and implications of right ventricular afterload in tetralogy of Fallot. Am J Cardiol. (2019) 124:1780–4. 10.1016/j.amjcard.2019.08.035 31586531

[22] Wiputra H Chen CK Talbi E Lim GL Soomar SM Biswas A. Human fetal hearts with tetralogy of Fallot have altered fluid dynamics and forces. Am J Physiol Heart Circ Physiol. (2018) 315:H1649–59. 10.1152/ajpheart.00235.2018 30216114

[23] Bodhey NK Beerbaum P Sarikouch S Kropf S Lange P Berger F. Functional analysis of the components of the right ventricle in the setting of tetralogy of Fallot. CircCardiovasc Imaging. (2008) 1:141–7. 10.1161/CIRCIMAGING.108.783795 19808531

[24] Wald RM Haber I Wald R Valente AM Powell AJ Geva T. Effects of regional dysfunction and late gadolinium enhancement on global right ventricular function and exercise capacity in patients with repaired tetralogy of Fallot. Circulation. (2009) 119:1370–7. 10.1161/CIRCULATIONAHA.108.816546 19255342 PMC2764308

[25] Wolter A Gebert M Enzensberger C Kawecki A Stessing R Degenhardt J. Outcome and associated findings in individuals with pre- and postnatal diagnosis of tetralogy of Fallot (TOF) and prediction of early postnatal intervention. Ultraschall Med. (2020) 41:504–13. 10.1055/a-0753-0008 30453353

[26] Arya B Levasseur SM Woldu K Glickstein JS Andrews HF Williams IA. Fetal echocardiographic measurements and the need for neonatal surgical intervention in Tetralogy of Fallot. PediatrCardiol. (2014) 35:810–6. 10.1007/s00246-013-0857-3 24352665

[27] Friedman K Balasubramanian S Tworetzky W. Midgestation fetal pulmonary annulus size is predictive of outcome in tetralogy of Fallot. Congenit Heart Dis. (2014) 9:187–93. 10.1111/chd.12120 23834770 PMC4304675

