# Supplementary material for: Ventricular Myocardial Deformation in Fetuses With Tetralogy of Fallot: A Necessary Field of Investigation
Source: Front Cardiovasc Med. 2021 Dec 10;8:764676. doi: 10.3389/fcvm.2021.764676 (PMC8708933; doi:10.3389/fcvm.2021.764676)
Supplement: Supplementary file 1 [file Table_1.DOCX]

| **Supplemental Table 1.**Inter-and intra-observer reproducibility | | | | |
| --- | --- | --- | --- | --- |
|  | **Variable** | **ICC (95%CI)** | **Bias±1.96SD** | **95%LoA** |
| Inter -observer | LV GLS | 0.84(0.48, 0.96) | -0.12±2.59 | (-2.71, 2.46) |
|  | RV GLS | 0.88(0.60, 0.97) | 0.23±2.96 | (-2.73, 3.18) |
| Intra-observer | LV GLS | 0.86(0.53, 0.96) | 0.01±2.70 | (-2.69, 2.71) |
|  | RV GLS | 0.87(0.56, 0.97) | -0.16±2.90 | (-3.07, 2.74) |
| ICC, intraclass correlation coefficient; CI, confidence interval; LoA, limits of agreement; LV GLS, left ventricular global longitudinal strain; RV GLS, right ventricular global longitudinal strain. | | | | |
